# Impact of resistance training status on trunk muscle activation in a fatiguing set of heavy back squats

**DOI:** 10.1007/s00421-020-04540-0

**Published:** 2020-11-18

**Authors:** David R. Clark, Michael I. Lambert, Chris Grigson, Angus M. Hunter

**Affiliations:** 1 School of Sport and Exercise Sciences, Faculty of Science, Liverpool John Moore’s University, Liverpool, UK; 2 Division of Exercise Science and Sports Medicine, Department of Human Biology, University of Cape Town, Cape Town, South Africa; 3 Physiology, Exercise and Nutrition Research Group, Faculty of Health Sciences and Sport, University of Stirling, Stirling, UK

**Keywords:** Back squat, Strength training, Neuromuscular, Electromyography, Trunk stabilizers

## Abstract

**Purpose:**

In this study we measured neural activation (EMG) in four trunk stabilizer muscles and vastus lateralis (VL) in trained and novice participants during a set of squat repetitions to volitional fatigue at 85% 1RM.

**Methods:**

Forty males were recruited into two groups, novice (NG: *n* = 21) and experienced (EG: *n* = 19), according to relative squat 1RM. Participants were tested twice to: (1) determine squat 1RM, and (2) complete a single set of repetitions to volitional fatigue at 85% 1RM. Relative squat 1RM; NG < 140% body mass, EG > 160% body mass. Neuromuscular activation was measured by EMG for the following: rectus abdominus (RA), external oblique (EO), lumbar sacral erector spinae (LSES), upper lumbar erector spinae (ULES) and VL in eccentric and concentric phase. Completed repetitions, RPE and EMG in repetition 1 and at 20, 40, 60, 80 and 100% of completed repetitions were analysed.

**Results:**

No group differences were found between number repetitions completed and RPE in repetitions to volitional fatigue at 85% 1RM. Neuromuscular activation increased significantly in all muscle groups in eccentric and concentric phase apart from RA in the eccentric phase. Trunk neuromuscular activation was higher in NG compared to EG and this was significant in EO, LSES and ULES in eccentric phase and LSES in the concentric phase. VL activation increased in both phases with no group differences.

**Conclusion:**

Trunk neuromuscular activation increases in a fatiguing set of heavy squats regardless of training status. Increased back squat strength through training results in lower neuromuscular activation despite greater absolute external squat loads.

## Introduction

Neuromuscular research into prime movers has been effective in advancing our understanding of the acute and chronic effects of squat training stimulus. For example, acute neuromuscular response to resistance training using the loaded free barbell back squat has contributed to our understanding of how to optimize adaptation in this exercise (Clark et al. 2012). Also, the effects of squat training and acquired back squat strength on activation of agonist muscles has been investigated (Häkkinen and Komi 1983; Häkkinen et al. 1998; Pick and Becque 2000). This research has shown a higher activation of agonist muscles in squat trained, compared to untrained subjects in squat one repetition maximum test (1RM) and during a single set of repetitions (reps) to failure at 85% 1RM (Pick and Becque, 2000). These findings can be explained by a higher agonist neural activation in trained compared to untrained participants.

Developing trunk stability has become increasingly recognised as a method of improving athletic performance and preventing injury (Wirth 2016). We reported that muscles of the lower limb have been investigated more than trunk muscles, i.e. hamstring and quadriceps activation data were reported more than any other muscle groups (Clark et al. 2012). Furthermore, we and others have investigated trunk stabilizer muscle activation in the back squat and established that activation is greater in concentric compared to eccentric phase and activation increases with increases in external load (Hamlyn et al. 2007; Nuzzo et al. 2008; Schwanbeck et al. 2009; Comfort et al. 2011; Aspe and Swinton 2014; Clark et al. 2016, 2020). We also showed that trunk muscle activation in loaded barbell squat was highest in the deepest range of both eccentric and concentric phases (Clark et al. 2020). The combination of external load, squat descent and transition to ascent represent the greatest challenge to trunk stability in the squat movement (Myer et al. 2014). Successful execution of the loaded barbell back squat is dependent on the capacity of the trunk stabilizers in maintaining the centre of force of the external load over the base of support throughout this movement. According the Myer et al (2014), ‘A more upright lumbar posture increases load onto lower extremity levers, which may reduce low back stress.’ This dynamic is supported by our data showing that regardless of squat training status, increases in external load result in greater trunk muscle activation (Clark et al. 2016). Recently, we demonstrated that trunk muscle activation in barbell squats at the same relative load is significantly lower in squat-trained versus untrained participants (Clark et al. 2020). This adaptation enables the prime movers to apply greater force with associated higher neuromuscular activation to achieve greater expressions of absolute (1RM) and relative squat strength (RM) by maintaining an upright trunk to ensure a vertical bar path over the midfoot. This suggests that increased trunk stability resulting from progressive load squat training is associated with greater neuromuscular efficiency in trunk stabilizer muscles and that loaded squats are an effective method of developing dynamic trunk stability.

It has been established that agonist activation increases during a fatiguing set to failure during moderate to heavy squats to offset loss of power (Pick and Becque 2000; Brandon et al. 2014). It was proposed that with fatigue from heavier loads, more motor units are were recruited including larger type II muscle fibres to compensate for the fatigued smaller type 1 fibres (Brandon et al. 2014). Stronger participants with greater maximal squat strength are capable of completing significantly more repetitions to failure (9.67 ± 0.91 reps) at the same relative squat load (85% 1RM) than weaker counterparts (7.14 ± 0.74 reps) (Pick and Becque 2000). In this study, trained subjects elicited higher muscle activation of quadriceps muscles across all repetitions to failure than weaker participants and this difference became significant closer to failure (Pick and Becque 2000). These authors concluded, higher agonist neuromuscular activation indicates greater motor unit discharge rate which explains greater absolute and relative strength and slower decay of strength in fatiguing repetitions to failure in stronger participants (Pick and Becque 2000).

We have previously shown that greater squat strength does not result in greater trunk muscle activation at moderate to heavy relative squat loads when compared to weaker participants (Clark et al. 2020). Maintaining an upright posture to ensure that barbell and external load remain over the base of support in the sagittal plane is dependent on tension developed in trunk stabilizer muscles (Myer et al. 2014). Our research demonstrates that participants who have developed a squat 1RM of ≥ 170% body mass are able to achieve necessary trunk muscle tension and stability in moderate to heavy squats at significantly lower muscle activation than participants with a 1RM of ≤ 140% body mass (Clark et al. 2020). These findings were determined in a test of three repetitions for each test load meaning the effect of fatigue on trunk muscle activation was not assessed. However, resistance training guidelines for muscular strength and power propose multiple repetitions of up to 12 per set, and trunk muscle activation in the squat under these conditions has not been investigated (Haff and Triplett 2016). Hence, progressive loaded free barbell back squat training results in increased absolute and relative load lifting capacity which is explained in part by more efficient neuromuscular function of the trunk stabilizers. How trunk muscle activation responds to a fatiguing set of back squat repetitions at a submaximal load has practical applications because this exercise is commonly used in training for strength and power development. The effect of back squat training status on trunk muscle activation under these conditions is also of interest. Therefore, the purpose of this study was to investigate acute trunk muscle activation in response to a single set of back squat repetitions to volitional fatigue at a heavy submaximal load and to compare the response of experienced and novice squat participants. We hypothesize that trunk muscle activation will increase in the repetitions to failure for all participants and that activation will be lower in the experienced group compared to the novice group.

## Methods

### Participants

Forty participants were recruited and divided into 2 groups according to relative back squat 1RM (Table 1). Participants with a minimum of 6 months back squat training were recruited from a range of university sports clubs and were free of injury and illness at time of testing. The novice group (NG) comprised of participants (*n* = 21) with a relative back squat 1RM of < 140% of body mass and experienced group (EG) comprised of participants (*n* = 19) with a relative back squat 1RM of > 160% body mass. Local institutional ethical approval for the study was obtained in accordance with the Helsinki declaration (2013). All participants gave informed written consent prior to testing.

**Table 1:** Mean (± SD) descriptive data for all participants by group

	Novice group (NG) *n*-21	Experienced group (EG) *n*-19	*P*	95% CI	Effect size rating
Age (years)	20.8 ± 4.0	24.6 ± 4.5	< 0.01	1.52–0.22	0.88 moderate
Body mass (kg)	79.4 ± 9.8	86.9 ± 13.2	= 0.06	127–0.00	0.64 small
Squat training age (years)	2.4 ± 1.1	6.4 ± 3.9	< 0.001	2.08–0.70	1.40 moderate
Strength training age (years)	4.0 ± 2.0	7.5 ± 4.1	< 0.01	1.74–0.41	1.08 moderate
Back squat 1RM (kg)	96.2 ± 15.6	156.3 ± 29.0	< 0.0001	3.44–1.73	2.57 large
Relative back squat 1RM (kg.kg^−1^)	1.2 ± 0.2	1.8 ± 0.1	< 0.0001	5.02–2.89	3.66 large
Test load at 85% SM (kg)	71.3 ± 12.5	121.3 ± 23.1	< 0.0001	3.54–1.83	2.68 large
Number of reps to failure	11.6 ± 4.1	9.8 ± 1.7	= 0.08	0.09–1.18	0.55 small
Rating of perceived exertion*	8.2 ± 0.4	8.5 ± 0.2	= 0.5	0.85–0.39	0.92 moderate

*kg * Kilogram, *1RM* 1 repetition maximum, *kg.kg-1* ratio of squat 1 repetition maximum to body mass in kilograms, *reps *repetitions, *CI *confidence interval

Level of significance *P* ≤ 0.05. *On completion of the single set to volitional fatigue at 85% SM

### Experimental design

Participants attended the laboratory for testing on two occasions. During the first visit, participants were briefed about the study. They signed consent, body mass was measured, training history data recorded, and they performed a back squat 1RM test. The second visit was scheduled within 3–7 days. During this visit, participants performed a single set of back squat repetitions to volitional fatigue at a submaximal load. Electromyography (EMG) for four trunk muscle sites and one lower limb site was measured during this set (Clark et al. 2016, 2020). An electro goniometer and linear transducer were used to measure kinematic data. This was synchronised with EMG data collection to determine eccentric and concentric phases for analysis.

### Laboratory visit 1: Back squat 1RM test

Participants completed a standardized warm-up, progressing from a range of compound body weight exercises to a barbell back squat warm-up. Back squat loads progressed to ten repetitions at 45%, eight repetitions at 55% followed by three repetitions at 65, 75 and 85% predicted 1RM. They had 2 min rest between sets. Thereafter single repetitions were performed to determine maximal 1RM load with correct depth and technique within five attempts, according to McGuigan (2016) (In, Haff and Triplett 2016). Testing was conducted by the primary investigator, an experienced strength coach and in accordance with established safety procedures for squat 1RM testing. Barbell position across the shoulders and foot position was standardised for each participant for all subsequent squat tests. All tests were performed in a safety squat rack (FT700 Power Cage, Fitness Technology, Skye, Australia) using competition approved barbell and discs (Eleiko, Sweden). Squat technique required participants to descend to where the tops of their thighs were horizontal or lower, followed by a controlled and continuous ascent to full hip and knee extension. Participants rested for 3 min between 1RM test efforts. Before departure, participants were briefed on the single set to volitional fatigue protocol scheduled for the second laboratory visit. They were also instructed on the procedure for failing safely in a repetition where they were unable to complete the ascent due to fatigue and given an opportunity to rehearse this. In this instance, participants were instructed to return the bar under control to the squat rack safety bars.

### Laboratory visit 2: Single set to volitional fatigue

Participants were weighed, screened for illness or injury and prepared for EMG capture. The sites of the five muscles and reference site were shaved, abraded and cleaned with an alcohol swab. Two adhesive electrodes (Ambu WhiteSensor WS, Ambu, Cambridgeshire, UK) were attached longitudinally at each site along the muscle fibre orientation with a 20-mm inter-electrode space according to SENIAM guidelines (Hermens et al. 2000). Electrodes were placed on the following muscle sites: rectus abdominus (RA), external oblique (EO) lumbar sacral erector spinae (LSES), upper lumbar erector spinae (ULES) and vastus lateralis (VL). A reference electrode was secured to the lateral malleolus of the participant’s right leg. Electrodes were connected to a BioNomadix 2 Ch. EMG Wireless Transmitter (BN-EMG2) to capture and transmit EMG data during the squat exercise. Transmitters were secured in harnesses: one on the upper back for posterior muscle sites (LSES and ULES), one on the mid-chest for anterior muscle sites (RA and EO) and one on the lateral calf for the VL and reference electrode. Cables and transmitters were secured to minimize artefact noise and prevent interference with execution of the exercise.

An electromechanical goniometer incorporating a high precision rotary potentiometer (6657 s-1–103, Bourns, Riverside, CA, USA), was attached to the right knee to measure knee angle through squat descent and ascent. The rotary potentiometer was placed at the centre of rotation of the knee and the goniometer fixed arm was attached to the lateral thigh by surgical tape. The actuating goniometer arm was attached by Velcro to a neoprene sleeve on the lateral calf and secured with surgical tape. The actuating arm incorporated three hinges to allow natural extension through the movement and a compact swivelling gimbal to accommodate small angular movement. The goniometer was manually calibrated to a fixed plastic protractor.

A threshold, established within Acqknowledge software (Version 4.4.2, Biopac Systems Inc, CA) produced a digital output when correct knee flexion was reached, indicating that required back squat depth was achieved or not. The required squat depth resulted in an audible sound and when this was not heard the set was terminated. Minimum knee flexion that corresponded to required back squat depth was established for each participant during the first barbell warm-up set and reinforced during subsequent loaded warm-up sets.

A single linear transducer (Celesco, PT5A, California, USA), fitted to the safety squat cage directly above the participant and attached to the middle of the barbell. The linear transducer measured displacement and time in eccentric and concentric phases of each completed back squat repetition. Linear transducer data were synchronized to root mean square (RMS) EMG data and used to demarcate back squat eccentric and concentric phases for analysis.

Participants completed the standardized warm-up as described previously. Calculating back squat loads according to system mass max (SM) is an established method in back squat research (Clark et al. 2016, 2020) and is based on the assumption that 89% of body mass is included in external load (Dugan et al. 2004). The remaining 11%, (i.e. shanks and feet) do not move vertically in squat exercise.

- SM = 1RM + (0.89 × body mass) (kg)
- External load = (SM × percentage of SM) – (0.89 × body mass) (kg).

Instructions for the single set to volitional fatigue protocol were repeated while participants rested for 5 min. They were instructed to perform continuous back squat repetitions while controlling descent and completing concentric phase as fast as possible. They were reminded to complete squats to parallel depth indicated by the audible signal, and to stop and replace the bar if they felt they were not be able to complete another full rep due to fatigue. Participants were also reminded of the procedure for failing safely where they were not able to complete a repetition ascent due to fatigue.

With a test load of 85% SM, participants completed the single set to volitional fatigue. Kinematic and EMG data were collected for warm-up sets at 65 and 75% SM and all repetitions of single set to volitional fatigue. On completion, rating of perceived exertion (RPE) was collected from each participant (Kraemer et al. 1987; Gearhart et al. 2002).

### Back squat data analysis

Rating of perceived exertion was recorded after three repetitions at 65 and 75% SM and single set to volitional fatigue at 85% SM. Total completed squat repetitions at 85% SM were recorded for each participant. Repetition 1 and identified repetition at 20, 40, 60, 80 and 100% of completed repetitions were identified and used to extract RMS data for analysis.

### RMS data analysis

**Fig. 1:**
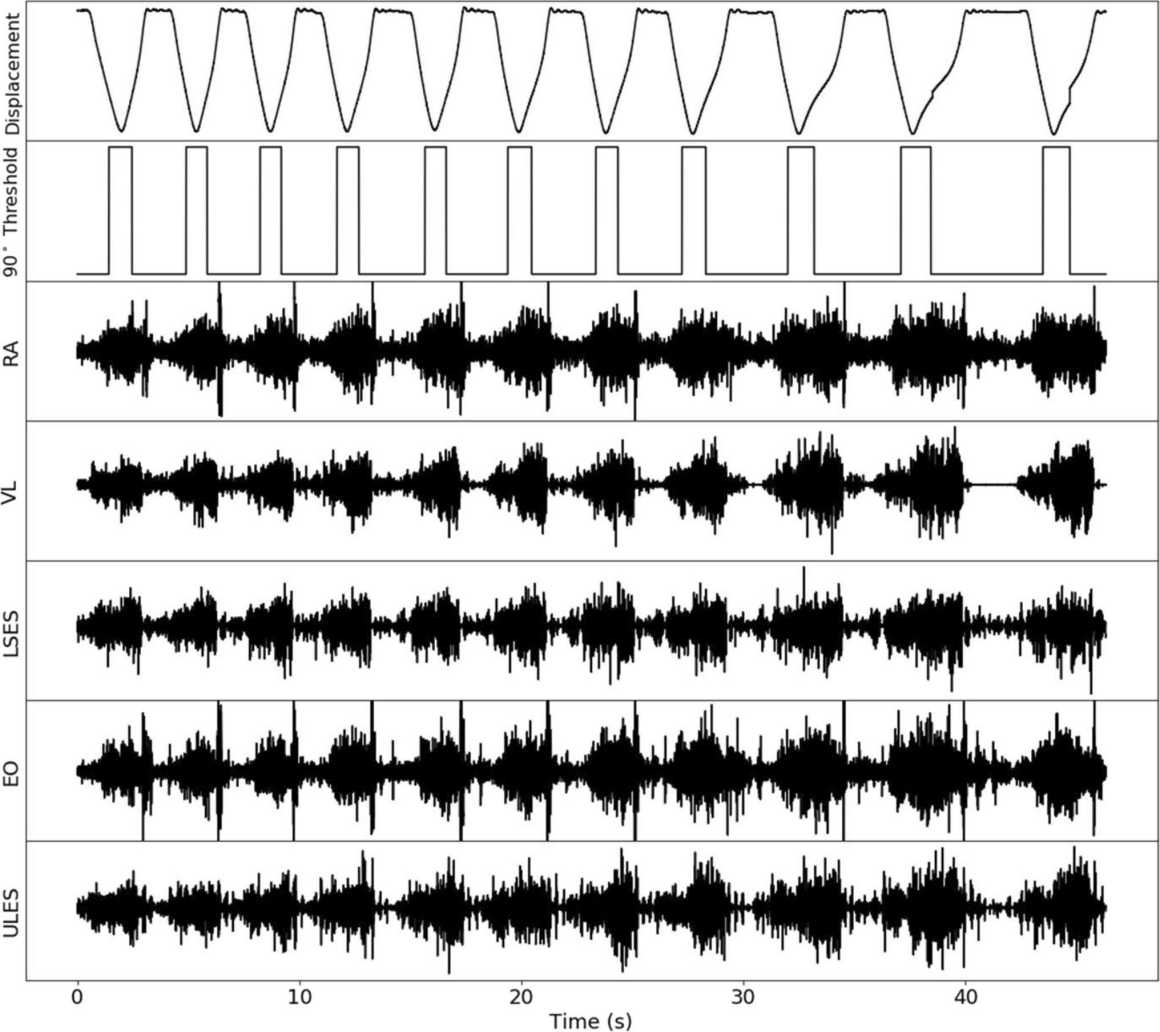
Typical trace of EMG and kinematic recordings for single set of repetitions to volitional fatigue. Displacement measured by linear transducer and 90° Threshold triggered by the electro goniometer measuring knee angle. *RA* rectus abdominus, *VL* vastus lateralis, *LSES*. lumbar sacral erector spinae, *EO* external oblique and *ULES* upper lumbar erector spinae

Electromyography was sampled at a rate of 2000 Hz, anti-aliased with a 500 Hz low pass filter in the Biopac MP150 system. The resulting signals were processed by applying an average root mean square (RMS) filter with a rolling 100-ms wide Bartlett window. The processed mean RMS was extracted for eccentric and concentric phase for each rep based on synchronised linear transducer data (Fig. 1). Spurious data points lower than 50% and higher than 400% RMS which were more than two standard deviation from the mean, were considered as outliers and removed prior to analysis. We have previously demonstrated moderately acceptable absolute (CV%, 12–20%) and relative (ICC, R = 0.60–0.79) reliability of mean RMS data for these trunk muscles in back squat exercise at similar loads (Clark et al. 2016).

Mean RMS for each muscle site in eccentric and concentric phase of each repetition were normalized to mean RMS of concentric phase of 65% SM and presented as mean percentage normalized RMS. Normalizing to a standardised relative submaximal contraction within the same exercise is well established (Albertus-Kajee et al. 2010, 2011) and we have demonstrated reliability of this method for assessment of trunk muscles in the back squat (Clark et al. 2016). It has been also demonstrated that submaximal dynamic contraction, not maximal isometric contraction, offer more reliable amplitude for EMG normalization of trunk muscles in healthy controls and patients with lower back pain (Balshaw and Hunter 2012).

Eccentric and concentric mean normalised RMS data for repetition 1 and identified repetitions at 20, 40, 60, 80 and 100% of completed repetitions, were extracted for analysis.

### Statistical analyses

Statistics were performed using GraphPad Prism version 7.04 for Windows. Unpaired two tailed *t*-test with Welch’s correction was used to determine group differences in participant and squat performance data. The magnitude of difference between groups was also expressed as effect size (Hedges effect sizes, ES) and interpreted according to criteria proposed by Rhea (2010) for strength training research in recreationally trained participants as follows:trivial ≤ 0.34, small 0.35–0.79, moderate 0.80–1.49 and large ≥ 1.5 (Cohen 1988; Rhea 2010). RMS and RPE data were analysed by two-way ANOVA with repeated measures and Sidak’s multiple comparisons test. Level of significance was set at *P* < 0.05. Data were analysed and presented for two groups: NG and EG.

## Results

### RMS data

**Fig. 2:**
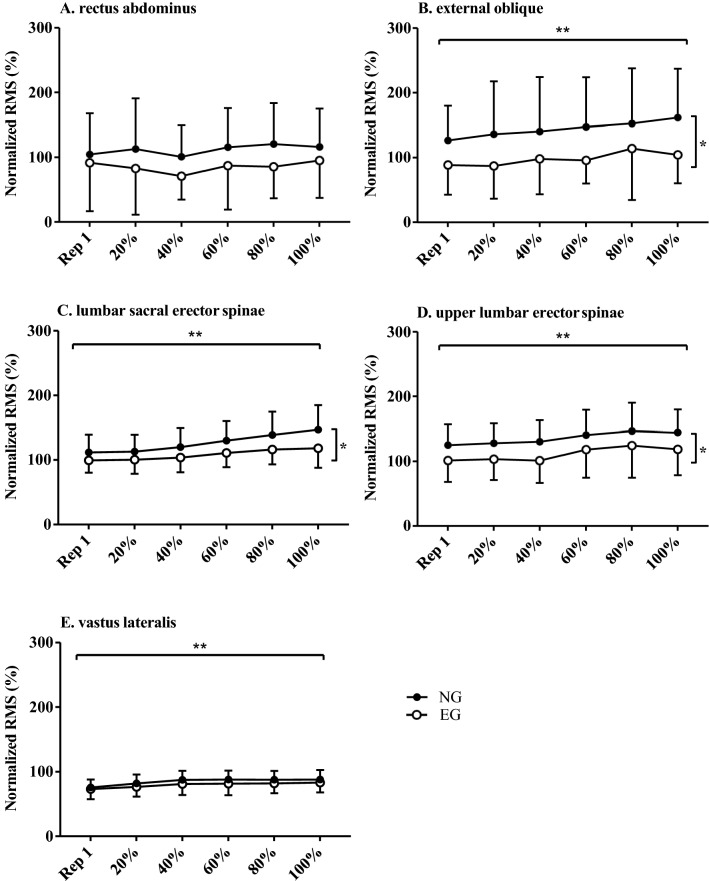
Normalized mean percentage RMS in eccentric phase for 2 groups (NG-novice group and EG-experienced group) at rep 1 and 20, 40, 60, 80 and 100% of completed reps to failure; ** Significant increase in RMS in both groups across all percentiles of reps to failure. *Significant differences between groups. (*RMS* root mean squared, rep-repetition)

**Fig. 3:**
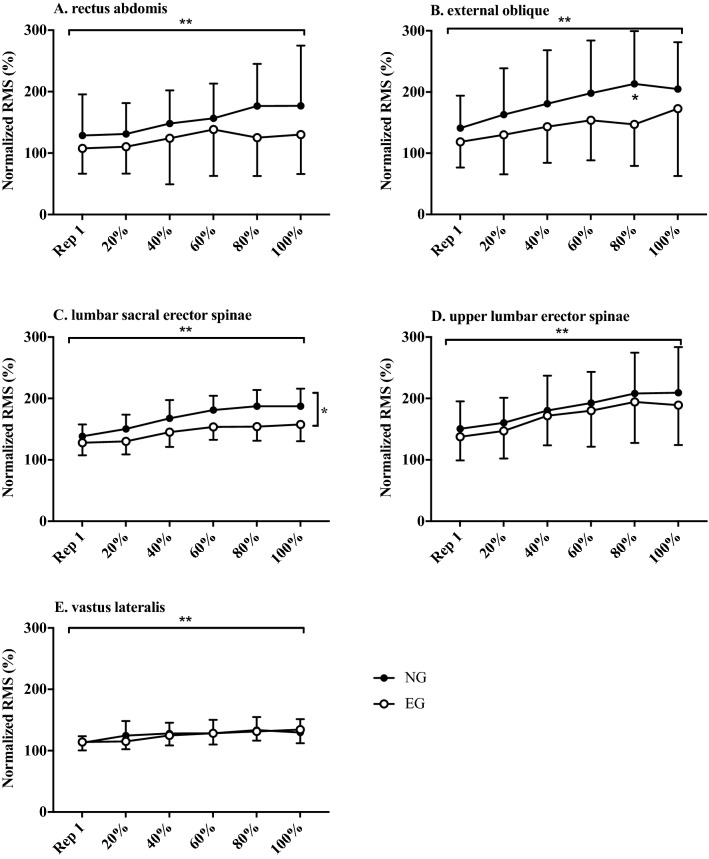
Normalized mean percentage RMS in concentric phase for two groups (*NG*-novice group and *EG *experienced group) at rep 1 and 20, 40, 60, 80 and 100% of completed reps to failure; ** Significant increase in RMS in both groups across all percentiles of reps to failure. * Significant differences between groups. (*RMS* root mean squared, rep-repetition)

Eccentric RMS increased significantly in repetitions to volitional fatigue in both groups and all muscle sites apart from RA (Table 2; Fig. 2). Eccentric RMS was significantly higher in NG group compared to EG in EO (F_1, 37_ = 5.4, *P* < 0.05), LSES (F_1, 38_ = 5.0, *P* < 0.05) and ULES (F_1, 36_ = 4.7, *P* < 0.05). Concentric RMS increased in repetitions to volitional fatigue in both groups for all muscle sites (Table 3; Fig. 3). Concentric RMS was significantly higher in NG compared to EG in LSES (F_1, 38_ = 13.5, *P* < 0.001).

**Table 2:** Mean percentage RMS at rep 1 and 20, 40, 60, 80 and 100% of completed reps to failure for all muscle sites and both groups in the eccentric phase

Eccentric		Rep 1	20%	40%	60%	80%	100%	95% CI	Reps to failure	Group ∆	Interaction
RA	NG	105	113	101	116	121	116	– 31.5 to 56,3	*P* = 0.2	*P* = 0.6	*P* = 0.7
	EG	102	91	89	106	97	112				
EO	NG	127	136	140	147	153	162	6.0 to 86.0	*P* < 0.0001	*P* < 0.05	*P* = 0.6
	EG	89	87	98	96	114	105				
LSES	NG	112	113	120	130	139	147	1.7 to 35.2	*P* < 0.0001	*P* < 0.05	*P* < 0.01
	EG	99	100	104	111	116	118				
ULES	NG	125	128	130	141	147	144	1.7 to 47.5	*P* < 0.0001	*P* < 0.05	*P* = 0.9
	EG	102	104	101	118	124	119				
VL	NG	76	82	87	88	88	88	– 3.4 to 13.8	*P* < 0.0001	*P* = 0.2	*P* = 0.9
	EG	73	76	81	82	82	83				

*RA* rectus abdominus, *EO* external oblique, *LSES* lumbar sacral erector spinae, *ULES* upper lumbar erector spinae, *VL* vastus lateralis, *NG* novice group, *EG* experienced group, *rep* repetition, ∆—difference. Level of significance *P* ≤ 0.05

### Back squat data

**Fig. 4:**
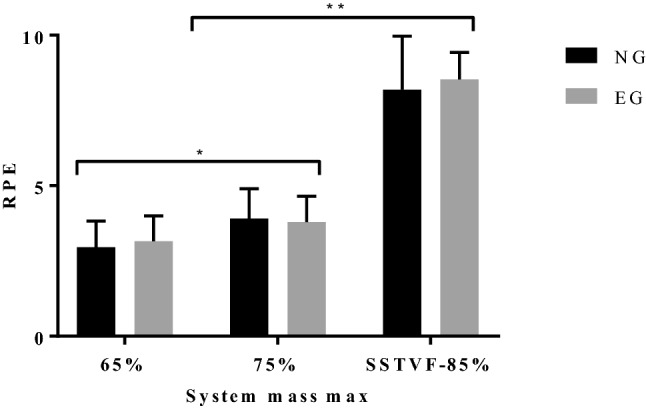
Mean rating of perceived exertion (RPE) for the novice (NG) and experienced group (EG) for three reps at 65 and 75% SM (system mass max) and single set to volitional fatigue (SSTVF) at 85% SM. *Significant difference between 65 and 75% SM (*P* < 0.01). **Significant difference between both 65 and 75% SM and SSTVF at 85% SM (*P* < 0.0001)

There were no group differences for the number of repetitions completed to volitional fatigue at 85% SM, nor for RPE on completion of the set to volitional fatigue (Table 1). There were significant group differences for squat training age (63%, *P* < 0.001), strength training age (47%, *P* < 0.01), absolute back squat 1RM (38%, *P* < 0.0001), relative back squat 1RM (32%, *P* < 0.0001) and absolute test load at 85% SM (41%, *P* < 0.0001). Effect sizes ranged from small to large. Mean RPE increased significantly (26%, *P* < 0.01) in the three repetitions at 75% SM compared to 65% SM and more than doubled (118%, *P* < 0.0001) after single set to volitional fatigue at 85% SM (Fig. 4).

**Table 3:** Mean percentage RMS at rep 1 and 20, 40, 60, 80 and 100% of completed reps to failure for all muscle sites and both groups in the concentric phase

Concentric		Rep 1	20%	40%	60%	80%	100%	95% CI	Reps to failure	Group ∆	Interaction
RA	NG	129	131	148	157	177	177	– 6.2 to 66.9	*P* < 0.0001	*P* = 0.1	*P* = 0.1
	EG	108	110	124	139	125	130				
EO	NG	141	163	181	198	213	205	– 4.5 to 83.0	*P* < 0.0001	*P* = 0.1	*P* = 0.1
	EG	119	130	143	154	147	173				
LSES	NG	138	150	168	181	187	187	10.8 to 37.1	*P* < 0.0001	*P* < 0.001	*P* < 0.01
	EG	128	130	145	154	154	158				
ULES	NG	151	160	180	193	208	209	– 19.6 to 46.5	*P* < 0.0001	*P* = 0.4	*P* = 0.9
	EG	138	147	172	180	194	189				
VL	NG	113	125	128	142	134	130	– 8.2 to 11.6	*P* < 0.0001	*P* = 0.5	*P* = 0.3
	EG	114	115	125	128	131	134				

*RA* rectus abdominus, *EO* external oblique, *LSES* lumbar sacral erector spinae, *ULES* upper lumbar erector spinae, *VL* vastus lateralis, *NG* novice group, *EG* experienced group, *rep* repetition, ∆—difference. Level of significance *P* ≤ 0.05

## Discussion

We found that neuromuscular activation of trunk stabilizer muscles and vastus lateralis increased in a single set of back squats to volitional fatigue at 85% SM. This was significant for all muscle sites and phases apart from RA in the eccentric phase. Neuromuscular activation was higher in NG compared to EG in EO, LSES and ULES in eccentric phase and LSES in concentric phase. Participants in each group were significantly different according to squat training experience (NG: 2.4 vs EG: 6.4 years), absolute squat 1RM test performance (NG: 96.2 vs EG: 156.3 kg), relative squat 1RM test performance (NG: 1.2 vs EG: 1.8 kg.kg^−1^) and test load at 85% SM (NG: 71.3 vs EG: 121.3 kg) representing the wide range of potential athletes and exercisers in the applied training environment. Despite this, there were no group differences in number of completed repetitions to failure (NG: 12 vs EG: 10 reps) nor RPE (NG: 8.2 vs EG: 8.5 RPE) after performing a single set to volitional fatigue at 85% SM.

### RMS in a single fatiguing set at 85% SM

Understanding acute neuromuscular response of trunk stabilizer muscles in a fatiguing set of barbell back squat repetitions has particular relevance for applied strength training. We know that activation of prime movers increases with acute fatigue during the squat to maintain power (Pick and Becque 2000; Brandon et al. 2014) and this study shows that the same applies for the trunk stabilizers. It is also established that stronger participants elicit increasingly higher prime mover activation across the duration of a squat set to failure (Pick and Becque 2000). In contrast, activation of trunk stabilizers was higher in weaker squat participants and this was significant in EO, LSES & ULES in the eccentric phase and LSES in the concentric phase. Furthermore, we did not find a group difference for vastus lateralis activation, as reported by Pick and Becque (2000). There were study design differences that may explain the contrast in findings. These authors normalized RMS in the single set to failure to RMS obtained in squat 1RM test conducted on a separate day, which required re-application of electrodes (Pick and Becque 2000). We normalized RMS in the fatiguing set to RMS in the concentric phase of the warm-up set at 65% SM in the same session according to Balshaw and Hunter (2012). Re-application of electrodes for between-day EMG capture increases EMG variance compared to within-day measurement (Dankaerts et al. 2004).

We have previously shown lower mean activation of trunk muscles in stronger compared to weaker participants during three repetitions of the squat at moderate (65 and 75% SM) and heavy (95% SM) relative loads (Clark et al. 2020). This was significant for all loads in eccentric phase and at 95% SM in the concentric phase. Absolute and relative squat loads were significantly higher in the stronger group who had a significantly greater mean squat training age of 4 years. It has been shown that increases in back squat strength from medium to long term progressive load squat training is achieved primarily through increased prime mover strength (Pick and Becque 2000), enhanced trunk stabilization at lower neuromuscular activation levels contributes to the improved squat performance. Trunk muscle adaptation ensures that trunk integrity is maintained at the increased absolute squat loads by preventing trunk flexion, especially in the more demanding concentric phase of the exercise. This study demonstrated that this efficiency, acquired through training translates to submaximal multiple repetitions to volitional fatigue, similar to the configuration in applied resistance training. This differs to the impact of training status on the response of prime movers under the same submaximal fatiguing conditions (Pick and Becque 2000). In both prime movers and trunk stabilizers, activation increases with fatigue; however, in stronger participants prime mover activation is greater than weaker participants throughout the set. Progressive strength training increases the capacity to activate a greater percentage of motor units in agonist muscles which results in increased absolute force producing capability (Brandon et al. 2014). This translates to greater relative submaximal force and greater relative performance in a fatiguing set of resistance training repetitions to failure. Under these conditions the role of trunk stabilizers is to maintain trunk stiffness and an upright posture to ensure that centre of mass remains over the base of support. This study confirms that acquired squat strength through training increases this capability with reduced trunk muscle activation.

The challenge placed on the trunk in both squat phases is predominantly to maintain extension of lumbar spine and avoid flexion in thoracic vertebral region (Myer et al. 2014). This corresponds with the reported high activation levels of the posterior trunk stabilizers in both phases of squats and in response to load increments. However, the role and therefore activation of RA and EO are not that obvious. Our findings demonstrating activation of both muscles (RA and EO) increase with load in both phases and is higher in the concentric phase, suggests that anterior and lateral trunk muscles contribute to stabilizing the spine and trunk in both the squat descent and ascent (Clark et al. 2016, 2019b, 2020). As a result, it is surprising that RA activation in this study did not increase in the eccentric phase during the repetitions to volitional fatigue.

In this study we found no group differences between experienced and novice participants in vastus lateralis RMS previously reported (Pick and Becque 2000), but our findings were similar in that vastus lateralis activation in both groups did increase for the duration of the fatiguing repetitions. A fundamental difference between the studies was the lower absolute squat strength of both our groups compared to Pick and Becque (2000). However, our study comprised more than double the number of participants a far wider range of squat strength and, therefore, more power. Furthermore, this is in agreement with Shimano (2006) who reported no difference in completed repetitions at a range relative intensities in the back squat based on resistance training status (Shimano et al. 2006).

Vastus lateralis eccentric RMS at 85% SM was lower than concentric RMS in the set to failure and lower than the submaximal reference value calculated in the concentric phase at 65% SM used for normalization. Eccentric vastus lateralis RMS in the set to failure was normalized to the mean concentric RMS for three repetitions at 65% SM during the warm-up. It is well established that concentric RMS is higher than eccentric RMS for any load of the squat exercise (Escamilla et al. 1998; Balshaw and Hunter 2012), and the leg press (Komi et al. 1987; Sarto et al. 2020). Sarto et al. (2020) demonstrated that eccentric RMS was the same for leg press at 70 and 80% 1RM and the only method of significantly increasing eccentric activation was by overloading the eccentric phase by 150% (Sarto et al. 2020). In the squat, we have previously shown that absolute unnormalized eccentric RMS (mV) is 42% lower than concentric RMS at 80% SM (Balshaw and Hunter 2012). The anomaly may be explained by higher activation in the concentric phase at 65% SM due to the novelty of the first loaded squat repetitions in the warm-up compared to fatigue-induced lower recruitment of larger motor units in the single set to failure at 85% SM.

Previous work from our laboratory showed that well-trained participants were not able to maintain initial barbell velocity in heavy load (85% SM) back squat as repetitions as sets progressed (Brandon et al. 2014). They demonstrated that this decrement in velocity and, therefore, power occurred despite a significant increase in activation of vastus lateralis. We demonstrated a similar increase in activation of the trunk stabilizer muscles also under these conditions; however, the effect of training status was the opposite, and stronger participants had lower activation than weaker participants at the same relative load. Or more importantly, reported adaptations in prime movers in response progressive load squat training is now supported by evidence of adaptations in trunk stabilizers to explain improved squat performance. Furthermore, we have shown that participants with higher squat strength produced significantly higher squat and countermovement jump heights with significantly lower activation of the trunk stabilizers during the concentric and flight phases (Clark et al. 2019a). Trunk stabilizer adaptation to progressive load squat training contributes significantly to improved squat performance by increasing trunk stability and stiffness at more efficient levels of neuromuscular activation.

### Squat performance

Percentage 1RM is a common method of manipulating acute resistance training intensity and 85% 1RM is reported to relate to approximately 6RM (Haff and Triplett 2016). We found an average of 11 repetitions to failure (Range: 5–22) at 85% 1RM back squat for all participants combined (NG and EG) with no significant difference between groups (Table 1). The wide range of repetitions completed to failure at 85% 1RM challenges the traditional relationship between percentage 1RM and RM, and it appears training status does not affect accuracy. Others, however, have found a significant difference in completed squat repetitions at 85% 1RM between trained (10 repetitions) and untrained groups (7 repetitions) (Pick and Becque 2000). They had fewer participants (Trained: *n* = 9 vs 19, Untrained: *n* = 7 vs 21), who were significantly stronger according to mean squat 1RM, (Trained: 184 vs 156 kg, Untrained: 120 vs 96 kg). The contributes to the growing challenge directed at the repetition maximum continuum (Haff and Triplett 2016; Fisher et al. 2020), specifically the accepted relationship between load calculated as a percentage 1RM and the repetition maximum method of load assignment (Hoeger Werner et al. 1990; Shimano et al. 2006). Our data also support evidence that training status does not influence the relationship between the relative 1RM and RM method of load calculation in the squat exercise (Hoeger Werner et al. 1990; Shimano et al. 2006; Mann et al. 2010).

## Conclusion

In conclusion, this is the first evidence that activation of trunk stabilizer muscles increases with fatigue during a single set of multiple repetitions to volitional fatigue in both eccentric and concentric phases in all muscles apart from the rectus abdominus in eccentric phase. Activation of trunk stabilizers is lower in squat trained participants under these conditions compared to those participants who are less trained. This research presents the first evidence describing the effect of squat strength on trunk stabilizer function in the squat. The evidence suggests that loaded free barbell squat repetitions to failure or volitional fatigue represents an effective method of developing dynamic trunk stability.

## Abbreviations

ANOVA: Analysis of variance
CI: Confidence interval
CV%: Coefficient of variation percentage
EMG: Electromyogram
EO: External oblique
ES: Effect size
Hz: Hertz
Kg: Kilogram
LSES: Lumbar sacral erector spinae
RA: Rectus abdominus
RM: Repetition maximum
RMS: Root mean square
RPE: Rating of perceived exertion
SM: System mass maximum
ULES: Upper lumbar erector spinae
VL: Vastus lateralis
